# Long-term use of lenalidomide and low-dose dexamethasone in Chinese patients with relapsed/refractory multiple myeloma: MM-024 Extended Access Program

**DOI:** 10.1186/s12885-016-2069-8

**Published:** 2016-01-28

**Authors:** Xin Du, Jie Jin, Zhen Cai, Fangping Chen, Dao-bin Zhou, Li Yu, Xiaoyan Ke, Xiao Li, Depei Wu, Fanyi Meng, Dena DeMarco, Jingshan Zhang, Jay Mei, Jian Hou

**Affiliations:** Guangdong General Hospital, Guangzhou, China; The 1st Affiliated Hospital, Zhejiang University, Hangzhou, China; Xiangya Hospital of Central South University, Changsha, China; Peking Union Medical College Hospital, Beijing, China; The 301 Hospital-Chinese PLA General Hospital, Beijing, China; Peking University Third Hospital, Beijing, China; Shanghai 6th Hospital, Shanghai, China; The 1st Affiliated Hospital of Soochow University, Suzhou, China; Nanfang Hospital of Southern Medicine University in Guangzhou, Guangzhou, China; Celgene Corporation, Summit, NJ USA; Department of Hematology, Shanghai Changzheng Hospital, Shanghai, China

**Keywords:** Relapsed/refractory multiple myeloma, Chinese patients, Lenalidomide, Low-dose dexamethasone, Dexamethasone

## Abstract

**Background:**

The efficacy and safety of lenalidomide plus low-dose dexamethasone (Rd) in Chinese patients with relapsed/refractory multiple myeloma (RRMM) was demonstrated in a phase 2, multicenter trial (MM-021). MM-024 was an Extended Access Program (EAP) that allowed responding patients in the MM-021 trial to continue to receive Rd, and to provide additional safety and efficacy data with longer follow-up.

**Methods:**

Chinese patients with RRMM who completed ≥1 year of Rd therapy in MM-021 and who remained progression-free under Rd entered the Treatment Phase of the MM-024 EAP, continuing Rd at the same dose and schedule. Patients in MM-021 who discontinued Rd treatment or progressed were allowed to enroll in the Safety Follow-Up Phase of the MM-024 EAP. Safety data, including the incidence of second primary malignancies (SPMs), were collected for ≥5 years from the time the last on-study patient enrolled in the MM-021 trial (primary end point). Efficacy outcomes (time to progression [TTP], progression-free survival [PFS], and overall survival [OS]) were secondary end points.

**Results:**

Median follow-up was 38.4 months for the safety population (*n* = 80) and 43.3 months for the treatment cohort (*n* = 41). In the safety population, Grade 3–4 adverse events (AEs) occurred in 60.0 % of patients; the most common grade 3–4 AEs were neutropenia (20.0 %), decreased neutrophil count (13.8 %), and anemia (11.3 %). There was no evidence of cumulative toxicity, and no patients discontinued Rd due to AEs; 2 patients had SPMs. In the treatment cohort, median duration of response was 35.1 months, median TTP was 36.9 months, and median PFS was 36.0 months; median OS was not reached due to the low number of deaths (*n* = 5).

**Conclusion:**

Long-term treatment with Rd has a predictable and manageable safety profile and provides sustained efficacy in Chinese patients with RRMM.

**Trial registration:**

China State Food and Drug Administration (SFDA) registration (CTA reference numbers: 209L10808; 209L10809; 209L10810; and 209L10811) and ClinicalTrials.gov Identifier: NCT02348528. First received January 23, 2015; last updated November 12, 2015; last verified November 2015; study start date September 2012.

## Background

During the past decade, significant improvements in survival outcomes have been achieved in multiple myeloma (MM) [1, 2]. Improved survival has been seen in all age groups up to 80 years of age. This success has been attributed in part to the introduction of novel therapies, which have greatly improved outcomes in patients with MM in both the frontline and relapsed/refractory settings [2, 3]. Despite these advances, virtually all patients with MM will eventually experience relapse or develop refractory disease and MM remains a fatal disease. Therefore, an unmet medical need remains for improved treatment options, particularly for patients with relapsed/refractory MM (RRMM).

In China, the combination of lenalidomide plus low-dose dexamethasone has recently been approved as a treatment option for patients with RRMM who have received ≥1 prior therapies. Approval was based on the results of the MM-021 registration trial, a large phase 2 study which evaluated the efficacy and safety of lenalidomide plus low-dose dexamethasone (Rd) in Chinese patients with RRMM [4]. This was the first study to evaluate Rd in this population. Low-dose dexamethasone was selected for use as opposed to high-dose dexamethasone due to the results of a previous randomized trial in newly diagnosed patients with MM that showed that Rd was associated with better short-term overall survival (OS) and less toxicity than lenalidomide plus high-dose dexamethasone (RD) [5].

In the single-arm MM-021 trial, 199 Chinese patients who received treatment with Rd yielded a high overall response rate of 47.6 % and a disease control rate (defined as at least stable disease) of 94.7 % [4]. Response rates were also high in the subgroups of patients with renal impairment and immunoglobulin D disease [4]. After a median follow-up of 15.2 months, the median progression-free survival (PFS) was 8.3 months (95 % confidence interval [CI] 6.5–9.8) [4]. The regimen also had an acceptable safety profile in this population. The most common adverse events (AEs) were hematologic and were manageable by dose adjustment of lenalidomide [4]. A venous thromboembolic event (VTE) was observed in only 1 patient [4]. Importantly, the pharmacokinetic profile of Rd in Chinese patients was similar to that reported in Caucasian and Japanese patients [6, 7].

The results of the MM-021 trial were consistent with the findings from two international phase 3 trials: one conducted in the United States (MM-009) [8] and the other in the European Union (MM-010) [9]. These studies demonstrated the efficacy and safety of RD as compared with placebo and high-dose dexamethasone (PBO + DEX) in RRMM patients. In a pooled analysis of the 704 patients who participated in the MM-009 and MM-010 trials, treatment with RD, compared with PBO + DEX, significantly improved response rate (60.6 vs 21.9 %, *P* < 0.001) and median OS (38.0 vs 31.6 months, *P* = 0.045), despite the fact that 47.6 % of patients randomized to PBO + DEX crossed over to the RD arm [10].

Few studies have assessed the long-term effects of treatment with lenalidomide plus dexamethasone in patients with RRMM, although available studies have consistently shown that long-term treatment was effective and well tolerated, with no increase in second primary malignancies (SPMs) [11–13]. The current study (MM-024) is both an extension of the MM-021 trial and a formal Extended Access Program (EAP), initiated to allow patients enrolled in MM-021 to continue treatment with Rd and, to collect efficacy and safety information. Here we report on the long-term safety and efficacy of Rd in Chinese patients with RRMM, based on data collected as part of the MM-024 EAP.

## Methods

### Patient eligibility

Patients were eligible to participate in the MM-024 EAP if they had participated in the MM-021 trial. Eligibility criteria for the MM-021 trial have been reported elsewhere [4]. Briefly, MM-021 patients had measurable Durie-Salmon stage II or III disease; had disease progression after ≥2 cycles of antimyeloma treatment or relapsed with progressive disease after therapy; had an ECOG performance status ≤2; and had adequate bone marrow reserve and liver and cardiac function [4]. Patients with renal failure requiring dialysis were excluded [4]. Patients were excluded from the EAP if they had serious hypersensitivity to lenalidomide or dexamethasone, or had previously discontinued lenalidomide therapy due to toxicity during treatment in the MM-021 trial or during the screening phase. Before participating in the EAP, all patients completed an informed consent document.

### Study design and treatment

**Fig. 1:**
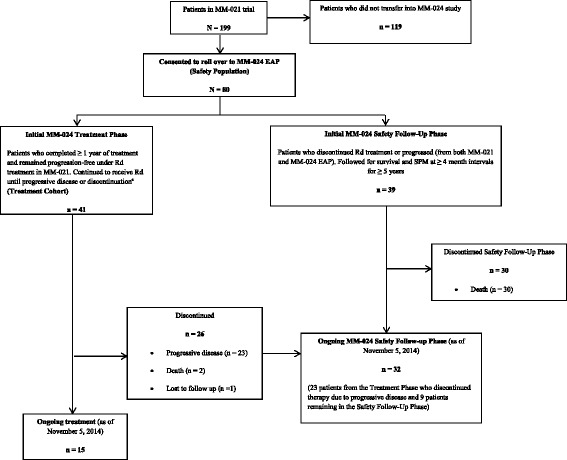
Overall study design and patient disposition at data cutoff (November 5, 2014). ^a^Patients received the same doses as in the MM-021 trial. *EAP* Extended Access Program, *Rd* lenalidomide plus low-dose dexamethasone, *SPM* second primary malignancy

The MM-024 EAP study (China State Food and Drug Administration [SFDA] registration [CTA reference numbers: 209L10808; 209L10809; 209L10810; and 209L10811]) is a multicenter, open-label EAP of Rd in Chinese patients with RRMM who participated in the MM-021 trial. The program consists of two phases, the Treatment Phase and the Safety Follow-Up Phase (Fig. 1). Patients who had completed ≥1 year of Rd therapy (from the start date of lenalidomide treatment) in the MM-021 trial, and remained progression-free under Rd treatment, were allowed to roll over to the Treatment Phase of the EAP to continue Rd treatment. Patients who had discontinued Rd therapy and were in the long-term follow-up phase of MM-021 were allowed to roll over to the Safety Follow-Up Phase of the EAP. Patients who discontinued Rd therapy during the Treatment Phase of the EAP were also entered in the Safety Follow-Up Phase of the EAP. The aim of the Safety Follow-Up Phase was to collect long-term safety data, including OS and incidence of SPM, in all consenting patients for ≥5 years from the time the last on-study patient enrolled in the MM-021 trial.

Patients in the Treatment Phase of the EAP received the same Rd regimen as in the MM-021 trial: lenalidomide 25 mg/day on days 1–21, and dexamethasone 40 mg/day on days 1, 8, 15, and 22 of each 28-day cycle for patients with normal renal function (creatinine clearance [CrCl] ≥ 60 ml/min); 10 mg/day for patients with mild-to-moderate renal function (CrCl ≥ 30 to < 60 ml/min); and 15 mg every other day for patients with severe renal insufficiency (CrCl < 30 ml/min). Any changes to the dose made in MM-021 for individual patients were retained in MM-024 EAP. Treatment continued until disease progression or discontinuation from study therapy for any reason (i.e., death, study withdrawal, lost to follow up) for a total duration up to 5 years (inclusive of 1 year of therapy in the MM-021 trial). Doses of lenalidomide and/or dexamethasone could be interrupted, modified, or reduced for drug-related AEs.

Patients at high risk of having a VTE continued to receive prophylactic anticoagulation therapy as they had in the MM-021 trial. Patients at high risk of a VTE could receive oral low-dose aspirin (70–100 mg daily) at the discretion of the treating physician. If aspirin was contraindicated, the use of low-molecular-weight heparin, Coumadin® (or an equivalent vitamin K antagonist), or other anti-thrombotic prophylaxis (according to hospital guidelines or physician preference was acceptable) was permitted for at least the remainder of the study treatment until disease progression.

The EAP was conducted according to the Declaration of Helsinki, International Conference on Harmonization guidelines for Good Clinical Practice, Ethics Committee procedures, and applicable local regulations. The study protocol was approved by the ethic committee boards of the following sites: Shanghai Changzheng Hospital, The First Hospital Affiliated of College Medicine, Zhejiang University, Xiangya Hospital Central South University, Shanghai 6th People’s Hospital, Peking University Third Hospital, NanFang Hospital, Changhai Hospital, The First Affiliated Hospital of Soochow University, The 301 hospital-Chinese PLA General Hospital, Peking Union Medical College Hospital, and Guang Dong General Hospital. Patients provided written informed consent prior to enrollment.

### End points and assessments

The primary end point was AEs. Safety data collected included the type, frequency, and severity of AEs, and their relationship to study drug. The incidence of SPMs, concomitant medication, and laboratory abnormalities were also recorded. The severity of AEs was graded using the National Cancer Institute Common Terminology Criteria for Adverse Events version 4.0.

Secondary end points were PFS, TTP, and OS. Survival and SPM were followed up at a minimum of every 4 months (±7 days). SPMs were reported as serious AEs, regardless of causal relationship to study drugs.

### Statistical considerations

The intent-to-treat (ITT) population contained all patients from the MM-021 trial who signed the informed consent of the EAP. The ITT population was used for efficacy analyses. The safety population consisted of all patients in the ITT population who received ≥1 dose of a study drug; this population was used for safety analyses. The treatment cohort was defined as patients in the Treatment Phase (i.e., those who had completed ≥1 year of treatment and remained progression-free under Rd treatment in MM-021 and continued to receive Rd as part of the MM-024 EAP). There was no statistical consideration in determination of sample size for the EAP.

## Results

### Patients and treatment

This report includes preliminary safety and efficacy results with a data cutoff date of November 5, 2014. The median follow-up from initial enrolment in the MM-021 trial was 38.4 months for the safety population and 43.3 months for the treatment cohort.

As of the cutoff date, a total of 80 patients were in the MM-024 EAP (safety population), 41 of these patients were receiving Rd when they entered the EAP (treatment cohort). Of the treatment cohort, at data cutoff, 15 patients were still receiving Rd, whereas 26 (63.4 %) patients had discontinued treatment. The primary reason for discontinuation was disease progression (*n* = 23); 2 patients died and 1 was lost to follow-up. For the safety population, the median duration of Rd treatment was 16.5 months (range, 1.8–48.1 months). In the treatment cohort, the median duration of Rd treatment was 23.3 months (range, 13.1–48.1 months), and 43.9 % (*n* = 18) of patients received >24.8 months (108 weeks) of Rd therapy. Of the 15 patients who remained on Rd treatment at data cut off, 9 were receiving the full dose of 25 mg/day of lenalidomide and 1 patient was receiving 10 mg/day (the full planned lenalidomide dose).

Patient baseline characteristics for the MM-024 EAP safety population and treatment cohort are presented in Table 1. For the safety population, median patient age was 59 years (range, 35–76 years), 28.8 % (*n* = 23) of patients were aged >65 years, and 65.0 % (*n* = 52) were male. The majority of patients in the safety population (80.0 %; *n* = 64) had Durie-Salmon stage III disease. In this cohort, 93.8 % (*n* = 75) of patients and 6.3 % (*n* = 5) of patients had Eastern Cooperative Oncology Group (ECOG) performance status scores of 0–1 and 2, respectively. In the treatment cohort, median patient age was 59 years (range, 47–74 years), 34.1 % (*n* = 14) of patients were aged >65 years, 61.0 % (*n* = 25) were male, and 78.0 % (*n* = 32) had Durie-Salmon stage III disease. In this cohort, 90.2 % (*n* = 37) of patients and 9.8 % (*n* = 4) of patients had ECOG performance status scores of 0–1 and 2, respectively.

**Table 1:** Patient baseline characteristics

Characteristic	Safety population (*n* = 80)	Treatment cohort (*n* = 41)
Age, years		
Median	59.0	59.0
Range	35.0–76.0	47.0–74.0
Age distribution, n (%)		
≤ 65 years	57 (71.3)	27 (65.9)
> 65 years	23 (28.8)	14 (34.1)
Sex, *n* (%)		
Male	52 (65.0)	25 (61.0)
Female	28 (35.0)	16 (39.0)
ECOG performance status score, n (%)		
0	31 (38.8)	19 (46.3)
1	44 (55.0)	18 (43.9)
2	5 (6.3)	4 (9.8)
Durie-Salmon stage, n (%)		
I	6 (7.5)	2 (4.9)
II	10 (12.5)	7 (17.1)
III	64 (80.0)	32 (78.0)
Number of prior antimyeloma therapies, n (%)		
1	9 (11.3)	6 (14.6)
2	13 (16.3)	10 (24.4)
3	12 (15.0)	5 (12.2)
4	12 (15.0)	6 (14.6)
≥ 5	34 (42.5)	14 (34.1)
Prior usage of bortezomib and thalidomide, n (%)		
Used thalidomide previously	59 (73.8)	28 (68.3)
Used bortezomib previously	57 (71.3)	31 (75.6)
Used neither thalidomide nor bortezomib	5 (6.3)	1 (2.4)
Used both thalidomide and bortezomib	41 (51.3)	19 (46.3)

*ECOG* Eastern Cooperative Oncology Group

In both populations, all patients had received prior antimyeloma treatment. In the safety population, the median number of prior therapies was 4 (range, 1–15); 71.3 % of patients (*n* = 57) had received prior bortezomib, 73.8 % (*n* = 59) had received prior thalidomide, and 51.3 % (*n* = 41) had received both. In addition, 6.3 % of patients in the safety population (*n* = 5) had undergone surgery, and 3.8 % (*n* = 3) had received radiation therapy. The median number of prior therapies in the treatment cohort was 3 (range, 1–11); 7.3 % (*n* = 3) of patients had undergone surgery and 2.4 % (*n* = 1) had received radiation therapy. A total of 75.6 % (*n* = 31) patients in the treatment cohort had received prior bortezomib, 68.3 % (*n* = 28) had received prior thalidomide, and 46.3 % (*n* = 19) had received both.

### Safety (primary end point)

Most patients (96.3 %; *n* = 77) reported ≥1 AE. The most common treatment-emergent AEs (TEAEs) of any grade were anemia (58.8 %), decreased neutrophil and white blood cell counts (47.5 and 32.5 % respectively), neutropenia (32.5 %), upper respiratory tract infection (33.8 %), fatigue (23.8 %), decreased platelet count (22.5 %), cough (21.3 %), pyrexia (20.0 %), and diarrhea (18.8 %).

The incidence of grade 3–4 AEs is shown in Table 2. Overall, 60.0 % of patients (*n* = 48) reported ≥1 grade 3–4 AE. The most common grade 3–4 TEAEs were neutropenia (20.0 %), decreased neutrophil count (13.8 %), and anemia (11.3 %).

**Table 2:** Grade 3–4 adverse events occurring in ≥3 % of the safety population

Adverse events	Safety population (*n* = 80)
Patient with ≥1 grade 3–4 adverse event	48 (60.0)
Neutropenia	16 (20.0)
Decreased neutrophil count^a^	11 (13.8)
Anemia	9 (11.3)
Pneumonia	7 (8.8)
Decreased white blood cell count	6 (7.5)
Leukopenia	5 (6.3)
Thrombocytopenia	5 (6.3)
Upper respiratory tract infection	5 (6.3)
Fatigue	4 (5.0)
Decreased platelet count	4 (5.0)
Hypocalcemia	3 (3.8)
Hypokalemia	3 (3.8)

All values *n* (%)

^a^Decreased neutrophil count refers to a fall in absolute neutrophil count since the last cycle, but not a low enough count to be considered neutropenia

Serious AEs were reported in 17.5 % of patients (*n* = 14), as shown in Table 3. The most commonly reported serious AE was pneumonia (8.8 %; *n* = 7). There were a total of 30 (37.5 %) deaths in the safety population; the most common causes of death were MM (*n* = 9), disease progression (*n* = 5), and lung infection (*n* = 4). In the treatment cohort, 5 deaths were reported; 2 deaths were during the MM-024 EAP Treatment Phase, and 3 deaths were after the patients entered the Safety Follow-Up Phase. Causes of death in the treatment cohort included plasmacytic leukemia, MM, cerebral infarction, treatment-emergent lung infection, and not specified (*n* = 1 for each). All patients in the safety population received anti-thrombotic prophylaxis in the form of aspirin; the most common concomitant medications were proton pump inhibitors (45.0 %; *n* = 36), bisphosphonates (40.0 %; *n* = 32), unspecified herbal and traditional medicines (33.8 %; *n* = 27), and fluroquinolones (28.8 %; *n* = 23).

**Table 3:** Serious adverse events in the safety population

Serious adverse events	Safety population (*n* = 80)
Patient with ≥1 serious adverse event	14 (17.5)
Pneumonia	7 (8.8)
Lung infection	2 (2.5)
Cerebral infarction	2 (2.5)
Bronchitis	1 (1.3)
Bronchopneumonia	1 (1.3)
Lobar pneumonia	1 (1.3)
Cerebral ischemia	1 (1.3)
Neutropenia	1 (1.3)
Cardiac failure	1 (1.3)
Cataract	1 (1.3)
Spinal compression fracture	1 (1.3)
Electrolyte imbalance	1 (1.3)
Multiple myeloma^a^	1 (1.3)
Deep vein thrombosis	1 (1.3)

All values *n* (%)

^a^Multiple myeloma was considered an adverse event when the disease worsened, but did not meet the study criteria for progressive disease

The average dose of lenalidomide was 21.7 mg (range, 6.3–25.0 mg). Median dose intensity (cumulative dose divided by overall treatment duration) was 18.0 mg and the median relative dose intensity (dose intensity divided by planned dose intensity) was 1.0. No TEAEs led to discontinuation of Rd. In the treatment cohort, TEAEs led to lenalidomide dose reduction in 14.6 % (*n* = 6) of patients, dose interruption in 41.5 % of patients (*n* = 17) and dose interruption and reduction in 14.6 % (*n* = 6) of patients. TEAEs in the safety population led to lenalidomide dose interruption in 42.5 % (*n* = 34) of patients, dose reduction in 7.5 % (*n* = 6) of patients, and both dose interruption and reduction in 18.8 % (*n* = 15) of patients. The most common AEs leading to lenalidomide dose interruption and/or reduction in the safety population were neutropenia (16.3 %), decreased neutrophil count (15.0 %), thrombocytopenia (6.3 %), upper respiratory tract infection (6.3 %), pyrexia (6.3 %), leukopenia (5.0 %), fatigue (5.0 %), pneumonia (5.0 %), and cough (5.0 %).

### SPM (primary end point)

Two SPMs were reported in the safety population: 1 patient had a duodenal tumor that was first reported during the MM-021 trial [4], and 1 developed nasopharyngeal carcinoma that was first reported during the Safety Follow-Up Phase in the MM-024 EAP study.

### Efficacy (secondary end points)

Secondary efficacy end points are shown in Table 4. In the safety population, median duration of response was 18.4 months (95 % CI 11.9–29.6), and time to progression (TTP) and progression-free survival (PFS) were both 13.8 months (95 % CI 11.2–20.4). For the treatment cohort, the median duration of response, TTP, and PFS were 35.1 months (95 % CI 25.5–38.2), 36.9 months (95 % CI 23.5–42.1), and 36.0 months (95 % CI 23.5–40.2), respectively. Median OS, based on Kaplan-Meier estimates, had not been reached at the data cutoff date, due to the low number of deaths for both the treatment cohort and safety population.

**Table 4:** Key efficacy outcomes

Efficacy outcome	Safety population (*n* = 80)	Treatment cohort (*n* = 41)
Duration of response, months	18.4 (11.9–29.6)	35.1 (25.5–38.2)
Time to progression, months	13.8 (11.2–20.4)	36.9 (23.5–42.1)
Progression-free survival, months	13.8 (11.2–20.4)	36.0 (23.5–40.2)
Overall survival, months	NE	NE

All values median (95 % CI)

*NE* not estimable

## Discussion

This study assessed the long-term safety and efficacy of Rd therapy in Chinese patients with RRMM. By extending treatment of the MM-021 trial, the MM-024 EAP has characterized the long-term safety of Rd in RRMM patients, about which little was previously known [11–13]. The results indicate that the Rd regimen has a predictable and manageable safety profile when given long-term in Chinese patients with RRMM. Overall, 60.0 % of patients had grade 3–4 AEs, and the most common grade 3–4 TEAEs were, as expected, neutropenia and anemia. No TEAEs led to treatment discontinuation; however, TEAEs led to lenalidomide dose interruption (42.5 %), reduction (7.5 %), or both (18.8 %) in the safety population. Median PFS and TTP in the treatment cohort were both approximately 36 months, and the median OS had not been reached at the data cutoff date due to the low number of deaths.

The safety results are generally consistent with the findings from the MM-021 trial [4] and indicate that Rd was better tolerated by the MM-024 EAP patients compared with those on RD in the MM-009 and MM-010 studies [8, 9]. With the extended follow-up of 38.4 months in the MM-024 EAP study (compared with 15.2 months in the MM-021 trial [4]), 60.0 % of patients had grade 3–4 AEs, compared with 69.8 % in MM-021 [4] and 85.3 % patients in MM-009 [8]. No marked increase in common grade 3–4 AEs was observed with longer follow-up; for most AEs, the incidence remained stable or decreased over time (anemia [26.1 vs 11.3 %], neutropenia [25.1 vs 20.0 %], thrombocytopenia [14.6 vs 6.3 %], and pneumonia [13.1 vs 8.8 %] for the MM-021 and MM-024 EAP studies, respectively) [4]. AEs led to lenalidomide treatment discontinuation in 19.8 % of patients in MM-009 [8], 9.0 % of patients in MM-021 [4], and 0 % in MM-024 EAP. Fewer patients required lenalidomide dose reduction and/or interruption due to AEs in MM-024 EAP compared with the MM-010 and MM-021 studies (18.8 vs 76.1 and 40.2 %, respectively) [4, 9].

The MM-009 and MM-10 studies [8, 9] demonstrated that RD was a superior treatment for RRMM patients compared to high-dose dexamethasone alone. Rajikumar et al. [5] demonstrated that in newly diagnosed MM patients, 1-year survival rates were significantly better for patients treated with Rd compared to those on RD; additionally Rd treatment was associated with significantly lower toxicity than RD. Therefore it was plausible that the superiority of the Rd regimen would be reflected in Chinese patients with RRMM, with a better safety profile compared to RD. Using Rd in the MM-021 trial addressed any safety concerns related to the use of high-dose dexamethasone in Chinese patients, and was consistent with the increasing use of lower doses of dexamethasone in combination with lenalidomide [5, 14].

Two SPMs were reported in the MM-024 EAP study (1 duodenal tumor, 1 nasopharyngeal carcinoma). The duodenal tumor was first reported in the MM-021 trial, before the patient entered the MM-024 EAP Safety Follow-Up Phase, and was not considered related to either lenalidomide or dexamethasone [4]. Thus, only 1 new SPM was reported as part of the MM-024 EAP study. This case of nasopharyngeal carcinoma was also deemed unrelated to treatment with lenalidomide or low-dose dexamethasone. Although there has been some concern regarding SPM risk when lenalidomide is given in newly diagnosed MM patients [15], the low incidence of SPMs in this study is consistent with other studies showing little or no increased risk of SPM with lenalidomide in RRMM patients [16].

Importantly, data from this analysis suggest that there are no long-term cumulative effects of lenalidomide-based therapy. No marked increase was observed in AEs typically associated with Rd therapy, such as neutropenia or infection, and there were no new AEs reported that were previously unseen with Rd therapy.

Efficacy outcomes were a secondary end point of the study, and compared favorably with those reported for the MM-021 trial. With longer follow-up and continued treatment with Rd, the treatment cohort of the MM-024 EAP had a median duration of response of 35.1 months and median PFS of 36.0 months, compared with 8.8 and 8.3 months, respectively in the MM-021 trial [4]. These results are promising, considering that 78.0 % of patients in the treatment cohort had Durie-Salmon stage III disease, 68.3 % had previously received thalidomide, 75.6 % had previously received bortezomib, and 46.3 % had previously received both thalidomide and bortezomib. These findings support the use of continuous treatment with Rd in RRMM patients.

Other studies have assessed the long-term safety and efficacy of lenalidomide plus dexamethasone in RRMM patients, using varying approaches. One analysis characterized patients from the MM-009 and MM-010 studies who achieved long-term benefit (median PFS ≥3 years) [11]. In this small subset of patients (*n* = 45) the response rate was 100 %. Notably, the incidence rate of neutropenia was lower in this subset than in patients with shorter PFS (13.9 vs 38.2 per 100 patient-years, respectively), whereas rates of SPM were similar in the two groups (1.7 per 100 patient-years in both groups) [11].

In a retrospective study of 50 patients with RRMM who received lenalidomide therapy for ≥2 years, the response rate was 96 %, and 74 % achieved a very good partial response or better [12]. Among this population, response rates were similar in those who received lenalidomide therapy for <3 years or ≥3 years. The safety profile was similar to that observed with shorter-term lenalidomide-based therapy: the incidence of neutropenia, thrombocytopenia and anemia were 16, 6, and 6 % respectively. Also, 20 % (*n* = 10) of patients experienced a VTE, and all but 1 were receiving thromboprophylaxis at the time of their VTE; the rate of SPM was low (2.0 %) [12].

Lastly, a retrospective study compared outcomes in patients with RRMM treated with RD for ≥12 months (*n* = 45) with those who discontinued therapy earlier (*n* = 10) [13]. Median OS was 42.9 months in patients treated for ≥12 months and 20.2 months in those treated for <12 months (*P* = 0.027). The main hematologic AEs were grade 3 or 4 thrombocytopenia (13 %) and leukopenia (9 %). Key non-hematologic toxicities included infection (25 % grade 3–4) and VTE (18 % any grade) [13].

Overall, the findings from these studies of the long-term safety and efficacy of lenalidomide plus dexamethasone in RRMM patients confirm the findings of the MM-024 EAP reported here.

## Conclusions

The results of this study indicate that long-term treatment with Rd is well tolerated in Chinese patients with RRMM. In this heavily pretreated population with advanced-stage disease, continued treatment with Rd led to improved response duration and prolonged PFS. Given the limited treatment options available in China for RRMM patients who fail multiple therapies, the availability of Rd will assist in meeting the need for improved treatment options. The results from this study provide further support for the long-term safety and efficacy of this regimen.

## Abbreviations

AE: adverse event
CI: confidence interval
EAP: Extended Access Program
ECOG: Eastern Cooperative Oncology Group
ITT: intent-to-treat
MM: multiple myeloma
OS: overall survival
PFS: progression-free survival
RD: lenalidomide plus high-dose dexamethasone
Rd: lenalidomide plus low-dose dexamethasone
RRMM: relapsed/ or refractory multiple myeloma
SPM: second primary malignancy
TEAE: Treatment-emergent adverse event
TTP: time to progression
VGPR: very good PR
VTE: venous thromboembolic event
